# Ultrathin Films of Cellulose: A Materials Perspective

**DOI:** 10.3389/fchem.2019.00488

**Published:** 2019-07-17

**Authors:** Eero Kontturi, Stefan Spirk

**Affiliations:** ^1^Department of Bioproducts and Biosystems, School of Chemical Engineering, Aalto University, Espoo, Finland; ^2^Institute of Paper, Pulp and Fiber Technology, Graz University of Technology, Graz, Austria

**Keywords:** cellulose derivatives, cellulose solvents, nanocellulose, spin coating, sensors

## Abstract

A literature review on ultrathin films of cellulose is presented. The review focuses on different deposition methods of the films—all the way from simple monocomponent films to more elaborate multicomponent structures—and the use of the film structures in the vast realm of materials science. The common approach of utilizing cellulose thin films as experimental models is therefore omitted. The reader will find that modern usage of cellulose thin films constitutes an exciting emerging area within materials science and it goes far beyond the traditional usage of the films as model systems.

## Introduction

Ultrathin films (thickness <100 nm) represent an important category of modern materials. Constant advances in deposition techniques as well as in micro and nanoscale patterning have significantly contributed in developing the functionality of thin films. Particularly in materials technology, ultrathin films play a major role in products like solar cells, sensors, and displays. It is obvious that the present research prospects reach far and wide.

Cellulose—the polysaccharide that is responsible for the structural scaffold of all plant fibers (Rosenau et al., 2018)—has a somewhat different history from many other materials concerning ultrathin films. Much of the research activity has been focused on utilizing thin films as experimental models, that is, as so-called model films (Kontturi et al., 2006). In this realm, cellulose thin films have been applied to study, for example, adsorption phenomena and water interactions with cellulosic materials—something that is difficult to directly deduce from morphologically and chemically heterogeneous natural fibers. Aside the modeling approach, the use of ultrathin cellulose films has recently expanded to materials applications, with proposed usage as biosensors and thermoelectric materials among others.

The aim of this literature study is to review the development with ultrathin films of cellulose for materials purposes roughly during the past decade. An exhaustive review (Kontturi et al., 2006) exists by one of the authors (EK) but, published in 2006, it is already grossly outdated. Moreover, the 2006 review focused nearly exclusively on the use of the films as model films chiefly because the aspect of materials applications was underdeveloped at the time. For this reason, we have here concentrated on different structures with cellulose ultrathin films and how one can utilize them as materials. In particular, a thorough account of different casting and post-treatment methods is given because of their importance precisely in the realm of materials science. Patterned structures, submonolayers, textures, and blends have emerged as an important part of the topic. We emphasize that the use of ultrathin cellulose films as model surfaces has been almost totally omitted in this review. This is not to undermine the significance of model films. The research on physico-chemical interactions with cellulose films has grown into a field with hundreds of literature accounts and the incorporation of those references would simply blow this review out of its proportions and overshadow the impact on materials science which stands as the objective here. In other words, the modeling approach warrants a review on its own. A good review already exists on surface forces in lignocellulosic systems (Österberg and Valle-Delgado, 2017) but a comprehensive review on all aspects of experimental modeling with ultrathin cellulose films is still missing.

The review is split in two sections. First, an account is given on various ways to deposit the cellulose films together with the background information and obvious challenges that are specific to cellulose. The second part is devoted to applications of ultrathin cellulose films in materials science. Some notions of most important references of the pre-2006 research are given, but the main focus is set on the research published after that, thus resulting in a markedly updated view on what one can achieve with cellulose films. We foresee that the review is not only useful for the cellulose specialist but that researchers from diverse fields can benefit and get ideas from its generic narrative.

## Film Deposition

**Figure 1:**
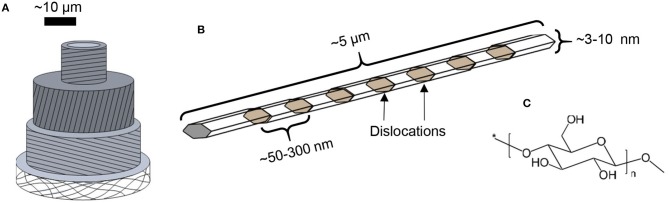
Schematic representations of **(A)** plant cell, **(B)** semi-crystalline cellulose microfibril, and **(C)** cellulose molecule.

Cellulose is the trivial name for poly(β−1 → 4-D-glucopyranose), consisting exclusively of linearly arranged anhydroglucose units (Figure 1). It is the structural component of all green plants. In fact, all biosynthesized cellulose occurs in the form of semi-crystalline microfibrils, long nanoscopic threads that form the structural scaffold of plant fibers (Figure 1; Nishiyama, 2009). The structural role of cellulose is the basis of its high strength and stiffness: individual microfibrils have been estimated to bear the tensile strength of ca. 2–6 GPa (Saito et al., 2013), and modulus at around 100 GPa (Eichhorn, 2012). Furthermore, plant fibers aim at longevity and durability and therefore, cellulose as its main structural component is relatively inert and stubbornly insoluble.

The sparse solubility of cellulose (Medronho et al., 2012) is an issue with thin film preparation. While the deposition of ultrathin inorganic films are dominated by vapor deposition techniques, thin films from soft materials are usually prepared by solution-based techniques. With ultrathin cellulose films, two deposition methods have dominated over others: spin coating and Langmuir-Blodgett (LB) or Langmuir-Schaefer (LS) deposition. In spin coating, high speed spinning of the solution on a substrate results in solvent removal and subsequently a solid film with a high degree of reproducibility (Norrman et al., 2005). In Langmuir-Blodgett deposition (Ariga et al., 2013), a solution of the to-be-coated substance is first deposited on a surface of an immiscible liquid (usually water), and the solvent is allowed to evaporate. In the second step, a monolayer of the floating substance on the liquid subphase is formed physically by a means of barriers (so-called Langmuir film) and finally, a solid substrate is dipped through the monolayer, resulting in ideally a monomolecular film on the substrate. The thickness of the film can be tuned by the number of dips, each contributing ideally to a rise of one monolayer in thickness. With both techniques, the intrinsic insolubility of cellulose poses problems which can be overcome or circumvented in three different fashions: (i) direct use of cellulose solvents, (ii) use of dissolving derivatives that can be regenerated to cellulose after film deposition, and (iii) use of colloidal dispersions of nanosized cellulose. The following passages will elaborate on each of these approaches.

### Film Deposition Directly From a Cellulose Solution

The prospect of applying a direct cellulose solvent for film deposition is attractive but it is far from an unproblematic approach. Aside from a class of relatively new solvents called ionic liquids, virtually all cellulose solvents are bicomponent or tricomponent systems involving a component that is solid at room temperature. These include mixtures of dimethylacetamide (DMAc) with LiCl (Dawsey and Mccormick, 1990), urea with aqueous NaOH (Cai and Zhang, 2005), or N-methylmorpholine-N-oxide (NMMO) with water (Rosenau et al., 2001). Obviously, if cellulose is cast from such solution into a solid film, the solid component from the solvent will also prevail in the film and it has to be removed by rinsing after the deposition.

**Figure 2:**
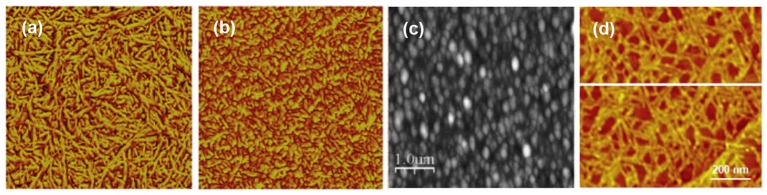
AFM images of cellulose films spin coated from a solution of **(a)** NMMO/water, 1 × 1 μm^2^ scan (Reproduced from Aulin et al., 2009 with permission from American Chemical Society), **(b)** DMAc/LiCl, 1 × 1 μm^2^ (Reproduced from Aulin et al., 2009 with permission from American Chemical Society) and **(c)** thiourea/NaOH/water, 5 × 5 μm^2^ scan (Reproduced from Yan et al., 2008 with permission from John Wiley & Sons, 2008); **(d)** individual cellulose molecules cast from a CED solution, 1 × 1 μm^2^ scan (copyright Elsevier 2007) (Yokota et al., 2007b).

Nearly all modern accounts with direct cellulose solvents utilize spin coating as the deposition technique (Generally, cellulose solvents are miscible in water and traditional LB-deposition with an aqueous subphase cannot therefore be used). Probably the most popular solvent has been NMMO/water or NMMO/DMSO which was introduced by the Wågberg group already in the early 2000s (Gunnars et al., 2002; Fält et al., 2004) while DMAc/LiCl as a casting solvent has also received some attention (Eriksson et al., 2005; Sczech and Riegler, 2006). A comprehensive survey by Aulin et al. (2009) on the crystallinity and morphology of different cellulose films revealed that the films spin coated from NMMO/water bore a semicrystalline structure with cellulose II, i.e., the order that arises from regenerated cellulose. The films spun from DMAc/LiCl, on the other hand, were almost completely amorphous. The films from both NMMO/water and DMAc/LiCl exhibited a fair degree of roughness, possibly due to the tracks left by the solid component after its removal (Figures 2a,b). Interestingly, the phase contrast image of the film deposited from NMMO/water shows clearly a fibrillar structure in contrast to the predominantly amorphous morphology of the film casted from DMAc/LiCl.

NMMO/water was also the solvent of choice when end-functionalized cellulose molecules were used for deposition (Yokota et al., 2007a). The idea was to attach a sulfur containing moiety (thiosemicarbazide) to the reducing end of dissolved cellulose molecules and subsequently expose the solution to a gold surface. The covalent bond, formed between sulfur and gold, aligned the cellulose chains parallel with each other, imposing a native cellulose I crystalline form, as analyzed by electron diffraction. Besides a recent account on thermally induced sol-gel transition from a DMAc/LiCl solvent (Wan et al., 2017), the end-tethering method is still the only form of regeneration that has led to cellulose I crystallites from solution. It is also a heavy indication of parallel alignment of cellulose I as opposed to anti-parallel alignment of cellulose II—a topic that has aroused substantial controversy over the years (Langan et al., 2001; Dinand et al., 2002; Kim et al., 2006).

Curiously, urea/NaOH/water, one of the new and relatively popular solvents for cellulose, has not been exploited in thin film deposition. However, its more obscure variant thiourea/NaOH/water has been used for spin coating cellulose with relatively similar morphology to previously published films from other direct solvents (Figure 2c; Yan et al., 2008). Ionic liquids are also a popular new class of cellulose solvents and one of them, namely 1-ethyl-3-methylimidazolium acetate (EMIMAc) diluted with DMSO, was used for thin film deposition by Kargl et al. (2015a). Films of thickness from submonolayer regime to hundreds of nanometers were reported.

A notable offshoot from the films deposited directly from a cellulose solution is the contribution by Yokota et al. (2007b) to cast submonolayers of single cellulose molecules (Figure 2d). The individualized single molecules were deposited by adsorption from a dilute cupriethylenediamine (CED) solution and subsequent evaporation of the solvent. Single molecular submonolayers have not been explored further with cellulose despite the undisputed popularity of the approach since the late 1990s in polymer science (Sheiko and Möller, 2001).

### Film Deposition via a Dissolving Cellulose Derivative

#### Monocomponent Cellulose Films

**Figure 3:**
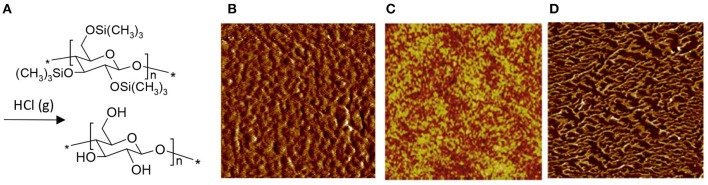
**(A)** Reaction scheme of TMSC conversion to cellulose with HCl vapor; AFM images of **(B)** spin coated cellulose film, regenerated from TMSC (Reproduced from Kontturi et al., 2003a with permission from American Chemical Society), **(C)** Langmuir-Schaefer deposited cellulose film, regenerated from TMSC (Reproduced from Aulin et al., 2009 with permission from American Chemical Society) and **(D)** Langmuir-Schaefer deposited cellulose monolayer on hydrophobized silica, regenerated from TMSC (Reproduced from Niinivaara and Kontturi, 2014 with permission from Royal Society of Chemistry). All images are 1 × 1 μm^2^ scans.

When utilizing a derivative as an aid to cast ultrathin films of cellulose, there is a single compound that dominates the research scene: trimethylsilyl cellulose (TMSC). Depending on its degree of substitution (DS), TMSC dissolves in many common organic solvents (Demeter et al., 2003). The TMSC solution can be effortlessly utilized in both LB deposition and spin coating and, once the film is cast, it can be hydrolyzed to cellulose by exposing it to fumes of aqueous HCl solution, as shown in the pioneering effort by Schaub et al. (1993) (Figure 3A). HCl vapor is able to fully hydrolyze the TMSC to cellulose without interfering much with the film morphology—something that would occur if the film was to be immersed in liquid HCl instead (Schaub et al., 1993). As a result, spin coated TMSC-based cellulose films (Figure 3B; Kontturi et al., 2003a,b) are much smoother than what can be achieved from direct deposition from a solvent (Figure 2). The LB deposited films from TMSC (Figure 3C; Holmberg et al., 1997) are highly smooth in any case due to the deposition technique.

The crystallinity of the TMSC-based cellulose films depends on the deposition technique. When LB deposition is used, the films turn out crystalline with the cellulose II polymorph (Aulin et al., 2009). With spin coating, on the other hand, the films are close to amorphous (Kontturi et al., 2011). Unlike many grades of bulk amorphous cellulose, the material in the TMSC-based ultrathin films does not start to crystallize when exposed to water (Kontturi et al., 2011).

The hydrolysis reaction from TMSC to cellulose in the 2D confinement of the supported ultrathin film received more attention in subsequent studies in the early 2010s. It was quantitatively shown that the thickness of spin coated TMSC contracted by about 60% upon hydrolysis, presumably due to the removal of bulky TMS groups and the onset of hydrogen bonding, largely prevented in TMSC but prevalent in cellulose (Kontturi and Lankinen, 2010). Simultaneously the density of the film, as monitored by x-ray reflectivity (XRR), increased from ~1 to ~1.5 g cm^−3^ which is close to the reported value for amorphous cellulose in bulk (Kontturi and Lankinen, 2010). Furthermore, an *in situ* quartz crystal microbalance (QCM) study (Mohan et al., 2012b) on the TMSC film hydrolysis showed similar results although the hydrolysis appeared to follow a two-phase kinetics contrary to the one-phase first order kinetics suggested by *ex situ* XRR. Another contemporaneous *in situ* study by total internal reflection (TIR) Raman spectroscopy also favored the two phase kinetic model (Woods et al., 2011). The possible reason behind the discrepancy between XRR and QCM/Raman data may lie in the scarcity of the measurement points in the XRR study. Meanwhile, small angle x-ray scattering (SAXS) measurements showed that substantial rearrangements occur in the cellulose film if the hydrolysis is continued further (up to 60 min) (Ehmann et al., 2015b). This was hypothetically ascribed to a greater mobility of shorter cellulose chains which are being formed by hydrolytic cleavage as a side reaction to the TMS group removal by acid vapor.

TMSC has also been used to prepare submonolayers films of cellulose, i.e., films where the amount of cellulose is so low that it does not exhibit full coverage over the substrate. When spin coated, the submonolayers appear as nanosized patches made of ca. 20–30 individual cellulose chains (Kontturi et al., 2005a). After LS deposition, on the other hand, the submonolayer morphology depends on the substrate used. In case of a hydrophobic, low surface energy substrate, the TMSC and subsequent cellulose submonolayers contract into fractals because of the entropic strive (Figure 3D; Niinivaara and Kontturi, 2014).

In the modern literature dominated by TMSC, another cellulose derivative to be used for cellulose thin film preparation is cellulose xanthate (CX) which is widely used for manufacturing viscose fibers and films and therefore a large scale commodity. Thin films were prepared by spin coating CX on silicon and gold substrates followed by regeneration using HCl vapor (Weißl et al., 2018). In contrast to TMSC, side products are not exclusively volatile but must be removed by a rinsing step using water. The obtained films are smooth and exhibit similar properties as the films derived from TMSC (e.g., with BSA adsorption).

#### Multicomponent Films

Because of the good solubility of TMSC in common solvents, it has been applied to prepare mixed or blended films with other polymers. Ultrathin films from polymer blends is a field that developed significantly during the late 1990s and early 2000s when atomic force microscopy (AFM) became a ubiquitous commodity in research laboratories worldwide (Xue et al., 2012). The idea behind polymer blend films is simple: when two immiscible polymers, dissolved in a common solvent, are cast onto an ultrathin film, phase separation occurs. The emerging phase separation patterns depend on the interactions between the polymers, their solubility in the relevant solvent, molecular weight, surface free energy, and the interactions between the polymers and the substrate. By simply spin coating two polymers from a common solvent, it is possible to create a large palette of textures as ultrathin films. The approach has been used, among others, to prepare anti-reflection coatings from polystyrene (PS)/poly(methyl methacrylate) (PMMA) blends (Walheim et al., 1999).

**Figure 4:**
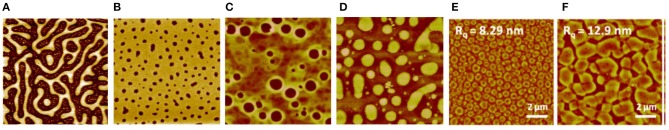
AFM images of blend films of cellulose, regenerated from TMSC with other polymers at following ratios: **(A)** TMSC/PS 2:1, 20 × 20 μm^2^ scan as in reference (Reproduced from Kontturi et al., 2005b with permission from American Chemical Society); **(B)** TMSC/PMMA 1:7, 5 × 5 μm^2^ scan (Reproduced from Kontturi et al., 2009 with permission from Royal Society of Chemistry); **(C)** TMSC/CTA 1:2, 5 × 5 μm^2^ scan (Reproduced from Taajamaa et al., 2011 with permission from Royal Society of Chemistry); **(D)** same film as in **(C)** but CTA has been removed with a selective solvent, 5 × 5 μm^2^ scan (Reproduced from Taajamaa et al., 2011 with permission from Royal Society of Chemistry); **(E)** TMSC/CSE 3:1, 10 × 10 μm^2^ scan (Reproduced from Niegelhell et al., 2017 with permission from American Chemical Society); **(F)** TMSC/CSE 1:1, 10 × 10 μm^2^ scan (Reproduced from Niegelhell et al., 2017 with permission from American Chemical Society).

With cellulose-containing materials, TMSC/PS films, subsequently hydrolyzed to cellulose/PS films, were the first ones to be introduced (Figure 4A; Kontturi et al., 2005b). Later on, TMSC/PS morphology was utilized to tune the hydrophobic/hydrophilic proportion on an ultrathin cellulose film (Nyfors et al., 2009) and to immobilize gold nanoparticles on cellulose films with high spatial precision (Taajamaa et al., 2013). Moreover, cellulose/PMMA films were introduced in the form of continuous PMMA layers with nano-sized cavities that bore disk-like cellulose at the bottom (Kontturi et al., 2009). The size of these cellulose-decorated cavities could be tuned by the original TMSC/PMMA ratio between 10–150 nm and the depth between 1–10 nm (Figure 4B).

TMSC was also used with cellulose acetate, to prepare ultrathin films of two distinct cellulose derivatives (Taajamaa et al., 2011). The resulting morphologies were peculiar with pores emerging that were exclusively in the cellulose acetate phase (Figure 4C). As usual, TMSC could be readily hydrolyzed to cellulose with HCl vapor but also cellulose acetate was hydrolyzed to cellulose with the use of ammonia vapor from aqueous NH_4_OH. After hydrolysis of one component, the other unhydrolyzed component could be removed by a selective solvent, leading to ultrathin cellulose films with unique morphological features (Figure 4D). Later on, the approach from 2D ultrathin films of TMSC/cellulose acetate was transferred to 3D morphologies of non-woven electrospun fiber networks (Taajamaa et al., 2012). Another study on two cellulose derivatives consisted of ultrathin films from TMSC and cellulose stearoyl ester (CSE) (Niegelhell et al., 2017). Either pillars or continuous networks of cellulose with CSE could be tailored according to the TMSC/CSE ratios (Figures 4E,F).

The initial concept of blend cellulose films was developed further with the introduction of cellulose/lignin ultrathin films, first by Rojas et al. (Hoeger et al., 2012) and later by the Spirk group (Strasser et al., 2016). Lignin, a non-linear macromolecule consisting of phenylpropyl units, is the second important macromolecule in landplants (Feofilova and Mysyakina, 2016). In their early effort, Hoeger et al. used TMSC with acetylated lignin to cast ultrathin films by spin coating and subsequently hydrolyzing them to cellulose/lignin films (Hoeger et al., 2012). In a later study, Strasser et al. utilized lignin palmitate with TMSC, followed by HCl hydrolysis which transformed TMSC into cellulose but left lignin palmitate intact (Strasser et al., 2016). In addition, cellulose/lignin palmitate blend films exhibited an unexpected behavior when exposed to bovine serum albumin (BSA) (Strasser et al., 2016). The lignin palmitate acted as a sacrificial layer which was emulsified when in contact with BSA solutions, as observed by quartz crystal microbalance with dissipation monitoring (QCM-D). These results were supported by AFM images showing that the LP domains shrank in width and height after BSA exposure. A similar mechanism was observed for cellulose—stearic acid (SA) blends (Niegelhell et al., 2017). In such materials, the stearic acid underwent self-organization and enriched on the surface after spin coating and subsequent regeneration. Similar as for the cellulose-LP blends, the SA was removed upon exposing it to BSA, as shown by surface plasmon resonance (SPR) spectroscopy.

**Figure 5:**
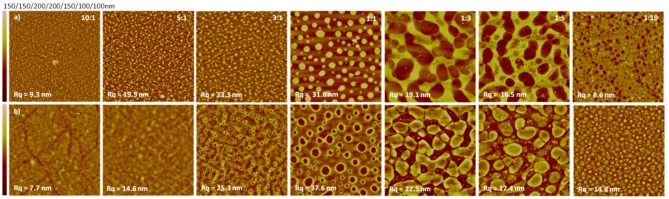
AFM images (size 10 × 10 μm^2^) of enzymatic degradation of PHB/cellulose thin films with PHB-depolymerase **(a)** or cellulase **(b)** (Reproduced from Niegelhell et al., 2016 with permission from American Chemical Society).

Concerning blend films incorporating TMSC, it is customary practice to optionally dissolve the other component after TMSC has been hydrolyzed to cellulose (Kontturi et al., 2005b). This is a routine found already in the seminal papers on polymer blend thin films, such as PS/PMMA (Walheim et al., 1997). With cellulose, however, a selective solvent for the other component is exceptionally easy to find because of the inherent insolubility of cellulose. Another way to approach selective removal of the other component is by specific enzymes. In this vein, Niegelhell et al. spin coated blend films from TMSC and poly(3-hydroxybutyrate) (PHB) and exposed them either to cellulose degrading cellulase enzymes or PHB degrading PHB-depolymerase (Niegelhell et al., 2016). The initial morphologies in TMSC/PHB blend films had TMSC forming protruding circular domains that shrank after their hydrolysis to cellulose (Figure 5).

#### Patterned Films via Lithography

**Figure 6:**
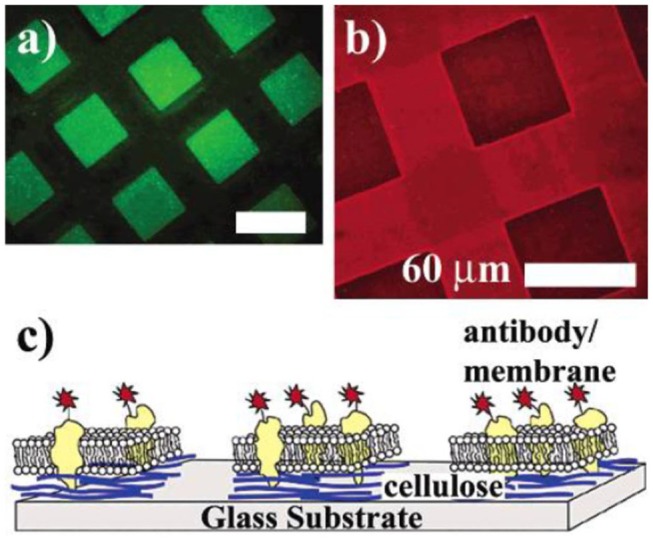
**(a)** Fluorescence image of cellulose micropatterns exposed to a solution of bovine serum albumin labeled with fluorescein isothiocyanate (FITC-BSA) after removing the photomask. FITC-BSA adsorbs only onto the ablated area (bare glass substrate). **(b)** Human erythrocyte membranes spread on cellulose microtemplates (without BSA treatment). After incubation, the cytoplasmic domain of Band 3 is identified with a monoclonal and a TRITC-labeled polyclonal antibody (inside label). **(c)** Orientation of the erythrocyte membrane after spreading. Erythrocyte ghosts selectively adhere and rupture only on the cellulose micropatterns, exposing their cytoplasmic side (Reproduced from Tanaka et al., 2004 with permission from American Chemical Society).

Ordered, geometrically regular patterns on ultrathin films are mainly achieved by lithographic techniques. The first report on micropatterned cellulose thin films was reported by Tanaka et al. (2004). They used hard UV lasers to decompose a cellulose film, thereby leaving pads of cellulose with a size of ~40 to 60 microns. The major idea of the patterning was to deposit cell membranes (i.e., human erythrocyte membranes) on the patterned cellulose thin films and to study the function of proteins in their native environment (Figure 6). Further they used the microtemplates to deposit microsomes from rabbit muscles. Both membrane patterns were stable for a period of over 1 week.

Another approach to induce patterns on cellulose is soft lithography in combination with enzymes (Kargl et al., 2013). This approach exploits the bioavailability of cellulose to enzymes capable of digesting it, i.e., cellulases. The approach works by protecting the cellulose by a micropatterned stamp. The accessible channels are filled with cellulase solution and the cellulose is digested. In such a way, cellulose micropatterns with feature sizes below 10 microns could be created. While this approach is elegant, it is quite laborious, setting a need for simpler routes.

One major step forward in patterning cellulose films was the use of masks protecting parts of the TMSC films during exposure to HCl. That way, regeneration occurs only in the accessible parts while the protected areas remain unaffected (Spirk et al., 2010). This procedure works on nearly any substrate although exact preparation procedures must be altered (e.g., use of 2-butanon as component in solvents) in order to deposit the TMSC onto specific polymer slides because standard solvents used for deposition may dissolve the substrate. These macroscopically structured slides were then used for a variety of applications (see section Applications Based on Surface Modification of Ultrathin Cellulose Films).

**Figure 7:**
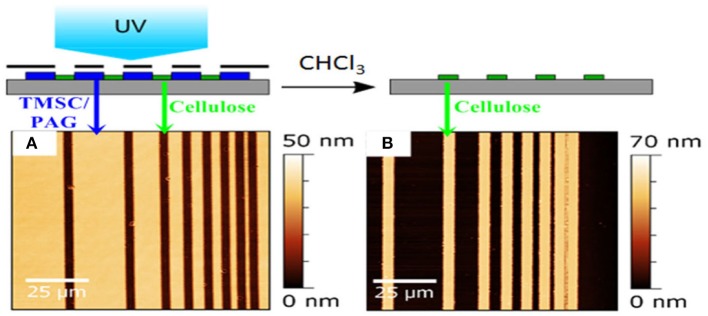
AFM images of TMSC ultrathin films using a photolithographic mask after irradiation **(A)** and rinsing **(B)**. The smallest structure has a size of ca. 1 micron and wavelengths below 365 nm have been excluded by an emissive filter to prevent degradation of cellulose during the illumination step. [Reproduced from Wolfberger et al., 2014 under the terms of the Creative Commons Attribution (CC-BY) license].

**Figure 8:**
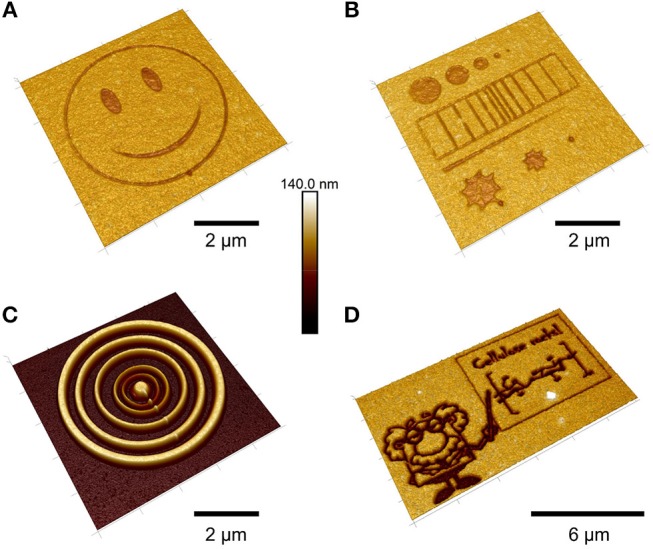
AFM height images of TMSC films treated with an electron beam **(A,B,D)** and subsequent development with toluene **(C)** [Reproduced from Ganner et al., 2016 under the terms of the Creative Commons Attribution (CC-BY) license].

While these approaches are highly reproducible and useful for the generation of large cellulose patterns, they have inherent limitations due to the size of the mask features that could be employed during HCl exposure. Therefore, concepts from polymer science were transferred to cellulose film patterning by using photo acid generators (PAG) (Wolfberger et al., 2014). PAGs are photolabile compounds, which decompose under illumination to generate acids. The main idea behind them is to induce a solubility change during the UV exposure which could be induced in principle by crosslinking, addition of functionalities or cleavage of functional groups. In the case of TMSC, the silyl groups are cleaved off in the illuminated areas, resulting in cellulose (Figure 7). The manufacturing of the resist works by addition of PAGs to the TMSC precursor, followed by spin coating the components and exposure to UV light under application of masks. Already after exposure, a clear step height difference is visible indicating that TMSC has been converted to cellulose. A following development step using the solvent used for preparation (to remove TMSC) yields micro and sub-microstructured cellulose thin films (Figure 8). Alternatively, the film can also be developed using enzymes (to remove the cellulose) and subsequent treatment with UV light leads to a positive type resist. Actually, this was the first cellulose based photoresist (see section Applications Based on the Internal Structure of Ultrathin Cellulose Films) (Wolfberger et al., 2015).

The feature size of the structures was further reduced to the real nanometer regime by a focused ion beam (Ganner et al., 2016). Usually, electron beams harm biopolymers rather fast to create carbon. Ganner et al. demonstrated that conversion to carbon can be overcome by exposing the films to the right dose, to merely induce regeneration of TMSC to cellulose. The influence of the dose was monitored by the enzymatic degradation of the structures and confirmed by ATR IR spectroscopy. Feature sizes down to 70 nm were created by this method.

### Film Deposition From Colloidal Nanocellulose Dispersions

With colloidal dispersions for cellulose film deposition, the idea is to circumvent the difficult solubility issue by omitting dissolution altogether. Here, it is important to use nanosized cellulose dispersions to retain the ultrathin status for the films. For example, microcrystalline cellulose consists of fiber fragment particles generally in the size range of tens of microns, which render them far too large for ultrathin film deposition (thickness <100 nm).

Nanosized cellulose—by and large—comes in two main types: cellulose nanocrystals (CNCs) and cellulose nanofibers (CNFs) (Klemm et al., 2011; Kontturi et al., 2018). Although technology for CNC isolation has existed since the mid-twentieth century (Rånby, 1951) and for CNF isolation since the early 1980s (Turbak et al., 1983), the real upsurge of nanocellulose research started after mid-2000s when research on renewables was ready to belatedly step into the nanotechnology bandwagon (Klemm et al., 2011; Kontturi et al., 2018). Contrary to most synthetic nanoparticles, both nanocellulose sorts are prepared by a top-down approach. CNFs are essentially isolated microfibrils obtained by disintegration of a delignified fiber cell wall (Abdul Khalil et al., 2014; Nechyporchuk et al., 2016). CNCs are, to simplify, cut CNFs that are attained via acid hydrolysis which breaks the semicrystalline microfibril into more or less individual crystallites by selective hydrolysis of the disordered segments in the microfibrils (Figure 1; Trache et al., 2017). It must be noted that CNCs are not isolated via CNFs but they can be acquired directly through hydrolysis of fibers.

#### Ultrathin Films of CNCs

CNCs are typically prepared by hydrolysis with concentrated sulfuric acid. The acid not only selectively cleaves the disordered segments in cellulose microfibrils: it also introduces sulfate half-esters on the CNC surface (Dong et al., 1998). The charged sulfate groups play an important role in stabilizing aqueous CNC suspensions with electrostatic repulsion. Even at relatively high concentrations, CNC suspensions are fluid dispersions, although slow separation into isotropic and liquid crystal phases can be found above a certain critical concentration (~10 wt.%) (Lagerwall et al., 2014).

**Figure 9:**
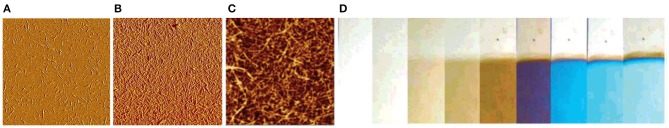
**(A)** AFM image of spin coated submonolayer of CNCs on TiO_2_, 5 × 5 μm^2^ scan (Reproduced from Kontturi et al., 2007 with permission from American Chemical Society); **(B)** AFM image of spin coated layer of CNCs with full coverage, 5 × 5 μm^2^ scan (Reproduced from Kontturi et al., 2007 with permission from American Chemical Society); **(C)** AFM image of LbL deposited CNF film, 1.5 × 1.5 μm^2^ scan (Reproduced from Wågberg et al., 2008 with permission from American Chemical Society); **(D)** photograph of LbL deposited CNF films of different thickness values, resulting in colored structures through optical interference (Reproduced from Wågberg et al., 2008 with permission from American Chemical Society).

The first account of ultrathin CNC films dates back to 2003 where Edgar and Gray applied spin coating to obtain smooth films that were stabilized with a mild heat treatment (Edgar and Gray, 2003). The treatment possibly removes water from between the CNCs, thus preventing re-dispersion when exposed to water. The phenomenon is possibly similar to hornification which is usually interpreted as a drying (and heat) induced irreversible aggregation of cellulose microfibrils in the fiber cell wall (Fernandes Diniz et al., 2004; Pönni et al., 2012). It is important to note that the mild heat treatment does not influence the sulfate groups on the surface of CNCs, as shown by Notley et al. who observed a large electrostatic component when probing the surface forces of CNC films (Notley et al., 2006). In any case, the method of spin coating and subsequent mild heat treatment has been used for preparing stable ultrathin films of CNCs ever since (Figure 9A).

Electrostatics brought in by the sulfated CNC surfaces are also helpful when controlling submonolayer deposition of CNCs. Anionic CNCs disperse neatly on a cationic TiO_2_ surface (Figure 9B; Kontturi et al., 2007), which has been later utilized when observing dimensions of individualized CNCs by AFM (Kontturi and Vuorinen, 2009). On anionic substrates, such as mica or silica, the CNCs tend to agglomerate in submonolayers, not enabling discerning of the individual CNCs.

Layer-by-layer (LbL) deposition is another means of film casting able to utilize charge on the CNC surface. Alternating layers of sulfated CNCs and cationic polyallylamine were introduced by Cranston and Gray (2006). Soon afterward, Podsiadlo et al. introduced LbL films of CNCs and polyethyleneimine which exhibited antireflective properties due to the suitable pore size distribution within the network of exceptionally long, tunicate-based CNCs (Podsiadlo et al., 2007). Since then, LbL films incorporating CNCs have emerged at regular intervals (Jean et al., 2008, 2009; Cerclier et al., 2010; Moreau et al., 2012; Olivier et al., 2012; Dammak et al., 2013; Olszewska et al., 2013; Azzam et al., 2014, 2015, 2017; Gill et al., 2017; Martin et al., 2017; Mauroy et al., 2018).

An account of all-cellulose films was presented by Niinivaara et al. (2016) who spin coated TMSC on a spin coated CNC layer and regenerated the TMSC to cellulose. Instead of appearing as two discrete layers, the TMSC was able to penetrate into the CNC network, forming a unique structure where CNCs were embedded in amorphous cellulose. The uptake of water vapor for these films exhibited an anomalous trend which did not follow the amorphous/crystalline ratio of amorphous cellulose and CNCs.

Besides spin coating, the accounts on alternative deposition techniques for CNC thin films are scattered. LB or LS deposition is not feasible for colloidal nanocellulose under the general setup of an aqueous subphase because nanocellulose dispersions themselves are virtually always dispersed in water. However, the Rojas group has reported LS films of CNCs by utilizing a cationic surfactant, dioctadecyldimethylammonium bromide (DODA) which easily forms a monolayer on water and complexes with CNCs that have been dispersed in the subphase (Habibi et al., 2010). Smooth ultrathin films with controlled thickness could be achieved from CNCs obtained from different sources, namely sisal, ramie, and cotton. The work was based on preliminary results on the same system, published few years earlier (Habibi et al., 2007).

Because nanosized cellulose is nearly always anisotropic, either rods (CNCs) or threads (CNFs), its orientation has been a pressing question, especially when used in composites as reinforcing materials. Orientation of CNCs in an ultrathin film would make a good precedent for orienting nanocellulose in larger matrices. In this vein, Hoeger et al. (2011) investigated orienting CNCs by a means of convective assembly—a method that has been highly successful in creating ordered layers of a variety of nanoparticles. It is based on capillary forces that work their way through the receding meniscus upon evaporation of a solvent. By the aid of a withdrawal plate to facilitate the movement of the meniscus, CNC films with >50% orientation could be formed. The technique was developed further by coupling convective assembly with electric field assisted shear assembly (Csoka et al., 2011).

#### Ultrathin Films of CNFs

Although CNFs as materials were introduced in the early 1980s (Turbak et al., 1983), it was not until 2006–2007 when their popularity surged due to several novel entries on preparation via mechanical disintegration with suitable pretreatments (Abe et al., 2007; Henriksson et al., 2007; Pääkkö et al., 2007) and an influential chemical pretreatment called TEMPO-mediated oxidation (Saito et al., 2006, 2007). The latter involves hypochlorite oxidation of cellulose catalyzed by 2,2,6,6-tetramethylpiperidine-1-oxyl radical (TEMPO) which is capable of selectively oxidizing the primary alcohol in the C6 position in cellulose This results in carboxylated cellulose microfibrils which can be effortlessly be individualized into CNFs because of electrostatic repulsion. Therefore, TEMPO-oxidized CNFs possess a high anionic charge density but indeed all CNF grades are at least mildly anionic due to the presence of hemicelluloses (mainly xylan) and oxidized structures on the microfibrils (Tenhunen et al., 2014; Tanaka et al., 2016).

The seminal problem with ultrathin film deposition concerning CNFs is their susceptibility to gel formation even at very low (~1 wt.%) concentrations (Fall et al., 2011). Gels are obviously not generally suitable for thin film deposition that are based on fluid suspensions or solutions. For example, the thickness control with spin coating does not work if one is not free to increase the suspension concentration of CNFs due to gelling. Even acquiring a film with full coverage from the dilute fluid suspensions is not straightforward. In the first successful account, Ahola et al. had to use 3-aminopropyltrimethoxysilane (APTS) as a high charge density cationic anchor for CNF films with full coverage (Ahola et al., 2008). Later on, the community has settled to use an adsorbed layer of PEI to anchor the CNFs (Figure 9C). Concerning submonolayer structures, Werner et al. created submonolayers by adsorbing CNFs on circular domains of printed cationic polyelectrolytes (Werner et al., 2008).

When slightly thicker films of CNFs in the ultrathin regime are in demand, LbL deposition with cationic polyelectrolytes is an option, although the films cannot be considered to consist of pure CNF. The Wågberg group has been particularly active with investigating LbL systems involving CNFs. In a fundamental contribution (Wågberg et al., 2008), CNFs with added anionic charge by carboxymethylation were deposited with either PEI, PAH, or poly(diallyldimethylammonium chloride) (PDADMAC). The thickness of the resulting LbL films with CNFs depended not only on the choice of polyelectrolyte but it could be controlled by electrolyte addition which affected the coiling of the polyelectrolyte during LbL. The different thickness values led naturally to varying degrees of optical interference, i.e., color formation within the films (Figure 9D). The build-up of CNF/PEI films was thoroughly investigated in a follow-up publication (Aulin et al., 2008). Continuation to the work was published in a paper on all-CNF films, resulting from an LbL deposition from anionic and cationically modified CNFs (Aulin et al., 2010). Moreover, copolymers of N-isopropylacrylamide and cationic (3-acrylamidopropyl)trimethylammonium chloride were utilized for a thermoresponsive LbL film with CNFs (Utsel et al., 2010).

Unicomponent CNF films with a thickness control over a wider range—without layer-by-layer approach—are feasible with electrophoretic deposition (EPD), as demonstrated for highly charged TEMPO-oxidized CNFs by Wilson et al. (2018). The work was partially based on an earlier study where EPD was used to prepare thick films of CNCs (Chen et al., 2014). Otherwise, TEMPO-oxidized CNFs have not been popular with thin films simply because of their instability after the deposition: TEMPO-CNFs tend to re-disperse because of their high charge density and tricks like heat treatment—which works well with CNCs of lower charge density—are not able to prevent the re-dispersion. Therefore, ultrathin films of TEMPO-CNFs have been reported mainly in such cases that have not involved liquid exposure after the film deposition, for example, when checking the film response to water vapor (Hakalahti et al., 2017). These films have been deposited by spin coating.

## Materials Applications of Ultrathin Cellulose Films

Unlike the vast influence imparted by nanocellulose on materials science (Kontturi et al., 2018), ultrathin cellulose films have been far less popular in materials applications. The flurry of activity during the present decade, however, indicates that ultrathin films in materials can be considered an emerging trend. The majority of accounts are based on, in one way or another, enhanced biomolecule immobilization on the film surfaces, focusing mainly on proteins and DNA. This is generally achieved by surface modification of cellulose films via covalent or non-covalent (chiefly adsorption) means. The other option is to utilize the internal structure of the films for, e.g., membranes or optoelectronics. The reader will notice that most of these applied accounts only hint toward genuine application and that the field is not as mature as that of film deposition elaborated in section Film Deposition.

### Applications Based on Surface Modification of Ultrathin Cellulose Films

The use of cellulose thin films as a support or active layer in materials development was originally driven from the biosensor perspective. Biosensors rely on specific interactions between a receptor and an acceptor. Such specific interactions are characteristic for biological systems and cover a wide range of biomolecules. Particularly protein-protein interactions have been in the focus of research activities on cellulosic surfaces. The main reason why cellulose is an excellent material for biosensor surfaces is its rather low non-specific interaction with proteins, while chemical modification (e.g., carboxylation, carbodiimide chemistry) can be easily accomplished. The low non-specific protein adsorption on cellulosic surfaces originates from the high degree of solvation of the cellulose molecules inside the thin film structures, thereby impeding the deposition of proteins having a large amount of water inside their structure as well. In order to realize a non-specific cellulose-protein interaction, water needs to be removed from the system, which is entropically unfavorable. In order to test surfaces toward non-specific protein adsorption, BSA is often used as a probe. It is well known that on neat cellulose thin films (e.g., from TMSC or CX), the amount of deposited BSA is very low under various conditions (Taajamaa et al., 2013; Weißl et al., 2018). Even close to the isoelectric point of BSA (4.8), where solubility is at its lowest due to a zero net charge of BSA, hardly any BSA adsorbs on a cellulose surface. However, the interaction of BSA with the cellulose surface can be increased if charged moieties, e.g., chitosan, cationic cellulose (Mohan et al., 2013b, 2014), cationic starch (Kontturi et al., 2008; Niegelhell et al., 2018), or carboxymethyl cellulose are introduced on the films. While the attachment of cationic polymers is irreversible due to electrostatic and entropic interactions, the irreversible deposition of carboxymethyl cellulose onto cellulose is far more intriguing, since a negatively charged polymer irreversibly adsorbs on a (slightly) negatively charged cellulose film. It has been shown that for related cases such as xyloglucans, energy is gained by conformational similarity between the cellulose and the xyloglucan chains. Therefore, it has been speculated that the same applies as the driving force for carboxymethyl cellulose (CMC) deposition on cellulosic substrates (Kargl et al., 2012).

**Figure 10:**
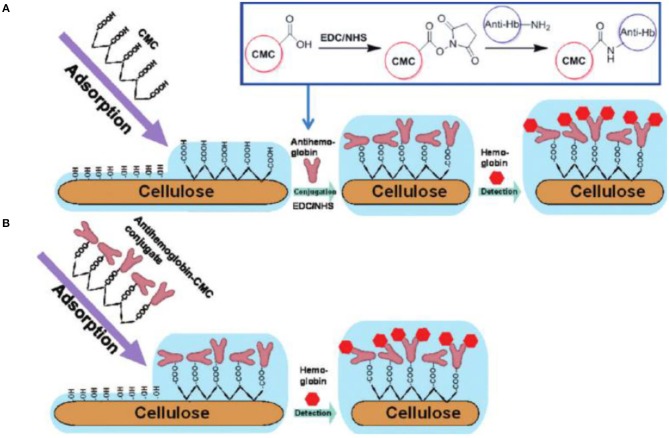
Preparation of Immunochemical Hemoglobin Assay on the Cellulose Surface. **(A)** Carboxymethyl cellulose (CMC) is irreversibly adsorbed on cellulose and then antibodies are covalently conjugated on CMC-modified cellulose and **(B)** antibodies are pre-conjugated with CMC and then the pre-conjugated conjugate is irreversibly adsorbed on cellulose (Reproduced from Orelma et al., 2012b with permission from American Chemical Society).

Since the charge of proteins depends on their pK_a_ value, they can be electrostatically deposited just by adjustment of the pH value. The group of Rojas showed in a benchmark study how physisorption of CMC and chitosan having different degrees of substitution affect the interaction of BSA and human immunoglobulin G (hIgG) with cellulose (Orelma et al., 2011). The idea was to use the charges of CMC and chitosan to tune the protein adsorption onto the surfaces, which was followed using a wide range of experimental techniques such as SPR and QCM-D. As expected, the highest amount of protein was determined close to the isoelectric points of the proteins (Orelma et al., 2011). Later, this concept was extended to cationic cellulose and trimethyl chitosans, where also the water content of the adsorbed protein layer was investigated by combination of QCM-D and SPR measurements (Mohan et al., 2013a, 2014). In the latter studies, different types of fluorescent labeled BSAs were additionally used to quantify the amount of deposited protein via fluorescent microscopy, showing a dynamic range of the modified cellulose film sensor probes from several μmol/L to some pmol/L. The rendering of the surfaces was accomplished via microspotting which enabled the automated fabrication of protein coated sensor slides on polymer substrates. The irreversible deposition of CMC on cellulose thin films was further used in other studies to anchor biomolecules onto the surfaces. Carboxylic groups on a surface enable the use of carbodiimide chemistry on cellulosic surfaces, which nowadays mostly involve the additional use of *N*-hydroxysuccinimide (NHS) esters to form a more stable intermediate with higher efficiencies in binding, particularly with NH_2_-rich molecules. One of the first attempts to exploit these concepts on regenerated cellulose thin films was carried out by Orelma et al. who chemically immobilized avidin on the surfaces using 1-ethyl-3-[3-dimethylaminopropyl]-carbodiimide (EDC) combined with NHS, i.e., so-called EDC/NHS chemistry (Orelma et al., 2012a). Avidin has a highly selective interaction with biotin (often referred to as vitamin B7). Therefore, biotinylation of different types of proteins has been a widely employed strategy to render surfaces with different types of proteins in a highly selective manner. The authors also showed that the immobilization of avidin as well as its coupling with biotinylated BSA and hemoglobin can be performed in high yields and proposed these films as generic substrates for antibody detection (Figure 10; Orelma et al., 2012b). Other examples that included the use of EDC/NHS chemistry to achieve binding of biologically active materials were related to blood specific applications. One of such applications involves the use of anticoagulant coatings for materials that come in direct contact with blood, i.e., catheters, blood tubings, and stents. In such applications, the blood coagulation needs to be prevented or at least delayed, which is usually realized by deposition of negatively charged polymers such as heparin or other sulfated polysaccharides. The Spirk group showed that cellulose thin films can be used as substrate for such an application. By deposition of CMC, followed by EDC/NHS chemistry or, alternatively, rendering the surfaces with PEI, simultaneous antimicrobial and anticoagulant silver and gold nanoparticles can be irreversibly deposited on such surfaces (Breitwieser et al., 2013; Ehmann et al., 2015a). The resulting functionalized surfaces showed significant prolongation of coagulation of blood serum compared to pure cellulose thin films.

**Figure 11:**
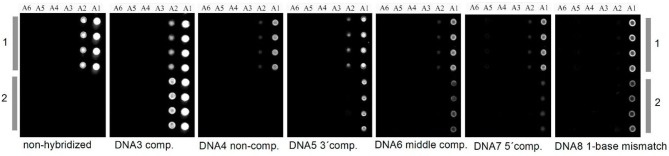
Carboxymethyl cellulose decorated cellulose thin films in DNA microarrays before and after hybridization (copyright Elsevier 2015) (Kargl et al., 2015b).

Concerning patterned cellulose layers created by masking and selective regeneration (see Figures 6–8), microspotting was used to deposit a further layer on top (e.g., CMC or cationic polysaccharides) in order to alter the surface for biomolecule immobilization. In the case of CMC, EDC/NHS chemistry was applied to anchor an ss-DNA strand (Kargl et al., 2013). These pads were then exposed to a complementary fluorescent labeled DNA strand, demonstrating high selectivity and affinity. The developed DNA sensor provided sensitivity down to 80 nmol/L of DNA (Kargl et al., 2013). The same authors later translated this technology into microarrays using the same approach, including optimization protocols (e.g., presence of ions or type of CMC) to improve the performance of the sensors (Figure 11; Kargl et al., 2015b). In a similar manner, protein sensor probes have been created by microspotting as already mentioned above (Mohan et al., 2013a, 2014) and functional conjugates using aminofluorescein as model compounds for proteins have been reported (Mohan et al., 2012a).

Blend films, such as those shown in Figures 4, 5, have been utilized with the aim to generate anti-biofouling surfaces with low non-specific protein adsorption. These films include PHB/cellulose, cellulose/cellulose stearate, and cellulose/cellulose hydroxypropylstearate blends (Niegelhell et al., 2016, 2017). For all films, there was a minimum in the BSA adsorption close to ratios of 1:1 and 1:3. In the case of PHB, hardly any BSA was adsorbing which led to speculations whether wetting effects of the films may cause this behavior. In such case, the one polymer is coating the other polymer via capillary forces, thereby forming a very thin layer on top of the whole film. Although there are some indications, force friction microscopy investigations did not reveal a clear correlation to support these speculations, at least for the cellulose/cellulose hydroxypropylstearate blend films. However, the blend films with the lowest surface free energy were also those with the lowest BSA deposition on the surface, i.e., the surface free energy of the blend film involving cellulose and a hydrophobic component was higher than that of pure cellulose. It is evident that such a difference must be caused by roughness effects whereas it seems that the surface configuration—periodicity of the structure and feature size—might be an underlying factor.

The group of Garnier studied the adsorption of various proteins on deuterated cellulose films (Su et al., 2016; Raghuwanshi et al., 2017a,b). In that case, deuteration was performed by supplying cellulose producing bacteria with deuterated nutrients (i.e., deuterated glucose) followed by trimethylsilylation and processing as for non-deuterated samples. Deuteration is accompanied by enabling better contrast in neutron scattering, thereby allowing for very precise layer thickness determination of the adsorbed layer. Horse radish peroxidase (HPR) as an important representative of peroxidases was investigated. HRP is an important protein since, like avidin, it is often used in biochemical assays in order to label proteins and to detect antibodies. In a similar manner, immunoglobulin G (IgG) antibodies were visualized by neutron reflectivity and compared to results from other techniques, such as QCM-D. The background of these studies was to evaluate the amount of IgG in respect to blood typing assays that were later reported on paper substrates.

Other methodologies to modify cellulose thin films by direct reaction on the surface have been recently introduced by the Stana-Kleinschek group. They showed that lysine moieties can be easily introduced on ready-made cellulose thin films by activation with N,N′-carbonyldiimidazole and subsequent aminolysis with lysine to yield a cellulose lysine carbamate film. In a similar manner, fuorates, and their aminated counterparts have been prepared (Elschner et al., 2016, 2018). These modifications have been developed to tune protein affinity to the surfaces, which was indicated by QCM-D.

In principle, a direct application of cellulose thin films is their use as a substrate for supported lipid bilayers (SLB) (Tanaka and Sackmann, 2005). SLBs serve as model systems for cell membranes to better understand membrane functions (e.g., transport, adhesion, signal transduction; Tanaka et al., 2001, 2004; Goennenwein et al., 2003). However, their manufacturing must consider several properties of the substrate they are deposited on. The support for the SLBs must be compatible to proteins and cells and should not induce denaturation of any component. Further, it should be amphiphilic (i.e., hydrophilic and hydrophobic) while being chemically stable toward the SLBs. Cellulose thin films fulfill these prerequisites, since their rather high degree of swelling leads to rather low non-specific interaction with biomolecules.

### Applications Based on the Internal Structure of Ultrathin Cellulose Films

This part of the review consists of accounts where the actual structure of the cellulose film has been utilized as a material—as opposed to the attempts that make use of the film surface, or modified surface, in the previous section. It is a more scattered collection of literature entries, resulting in a more inconsistent and uneven narrative.

As already mentioned, the micropatterned films created with the aid of PAGs were utilized as photoresists (see Figure 6; Wolfberger et al., 2014, 2015). A negative type resist with a thickness of 32 nm was shown to be efficient as dielectric gate material in thin film transistors. Performance indicators showed that gate leak currents as well as the impedance of the films feature excellent values.

Another contribution by the Spirk group focused on cellulose—PHB films (as in Figure 5) which were used to create bioorthogonally degradable films to realize so called bioresists (Niegelhell et al., 2016). These studies were further accompanied by fast scan AFM imaging providing videos of the enzymes (PHB depolymerase and cellulases) in real time. Additionally, the results were corroborated by SPR spectroscopy. Feature sizes between nano and micrometer size can be obtained depending on the ratio of the two polymers in the films.

The internal addition of precursors or functional molecules allows for further applications of cellulose based thin films. Here, the solubility of TMSC in organic solvents is exploited which allows for addition of either drugs, nanoparticle precursors, or even nanoparticles. For instance Maver and coworkers showed that cellulose thin films, doped with diclofenac, exhibit a similar release profile as with fiber treated samples (Maver et al., 2015). Since drug release studies on fibers are very laborious and time consuming, the use of cellulose thin films was proposed as a viable alternative to these tests. Furthermore, Parra et al. incorporated hydroxygallium phthalocyanine into the cellulose structure. That way, they could come up with a gas sensor capable to detect 0.1 ppm of oxygen which was better than just immobilizing the phtalocyanine alone (Parra et al., 2007). A different system was introduced as a humidity sensor probe, obtained by using a ternary system composed of alternating layers of cellulose, polyvinyl alcohol and poly(N-vinylcarbazole). Seven trilayers of these polymers formed a photonic crystal which showed a response toward alterations in humidity levels (Manfredi et al., 2016).

**Figure 12:**
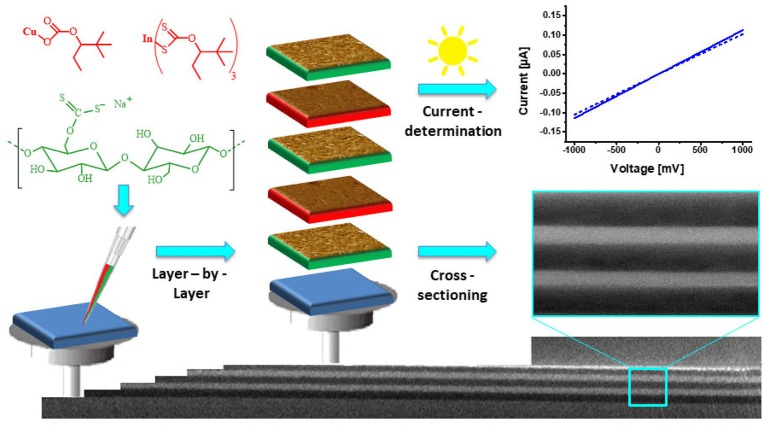
Schematic illustration of the preparation of cellulose/CuInS_2_ multilayer films and their characterization using cross-sectional SEM (Reproduced from Weißl et al., 2019 with permission from Elsevier).

Carrying on with the theme of incorporating components into the films, a variety of nanoparticles has been deposited on or in cellulose thin films. One strategy to create nanoparticle rendered thin films is to add a precursor to the TMSC solution, deposited by spin coating on a surface and either converted to the nanoparticle before or after regeneration of the TMSC to cellulose. Bismuth based nanoparticles have recently gained attention due to their potential use as new contrast agents for magnetic resonance imaging (Gösweiner et al., 2018). Such nanoparticles can be created from labile bismuth compounds such as BiPh_3_, and from bismuth xanthates that are added to TMSC before processing. While BiPh_3_ can be degraded by UV light to elemental Bi nanoparticles in the low nanometer range (Breitwieser et al., 2015), the xanthates are converted to the bismuth sulfides via the Chugaev reaction (80 nm length, 25 nm width) (Reishofer et al., 2017a). This procedure works also for other metal sulfides and in a similar way CuInS_2_ nanoparticles (diameters of few nm) have been created in a TMSC matrix. This was exploited in optoelectronic devices (a nanocrystal thin film solar cell) with good performance and a photocurrent efficiency of ca. 1% (Reishofer et al., 2017b). The xanthates can be also deposited as an individual layer in the surface of the cellulose films. This was accomplished on cellulose thin films from cellulose xanthate where alternating multilayers of cellulose and CuInS_2_ with adjustable thickness were created. Upon exposure to UV light, the CuInS_2_ layers generated photocurrents (Figure 12; Weißl et al., 2019). In another attempt with inorganic materials, Nissinen and coworkers studied the formation of calcium sulfates in/on cellulose thin films spin coated from NMMO in an industrial context. They pretreated the cellulose thin films with Ca^2+^ ions and exposed the films to concentrated solutions of Bassanite (calcium sulfate hemihydrate). They observed that depending on the activation procedure the rate of crystallization can be controlled (Nissinen et al., 2013, 2014). In a recent report, cellulose thin films were decorated with different nanodots [silicon-dots (SiDs), carbon-dots (CDs)] or nitrogen-doped carbon dots (N-CDs). The authors propose the use of these nanodots in biomedical applications due to their non-toxicity compared to other frequently used materials (Cuevas et al., 2019).

A multilayered structure with TEMPO-CNFs and ZnO was achieved by consecutive adsorption of CNFs and atomic layer deposition (ALD) of ZnO (Jin et al., 2017). These hybrid films were envisaged as thermoelectric materials because the thermal conductivity of the films could be controlled with the insulating cellulose layers in between the ZnO layers.

On a different note, the Peinemann group published a series of papers on thin film membranes based on cellulose thin films (Puspasari et al., 2016, 2018a,b,c). They used either crosslinking (e.g., by glutaraldehyde) or blending with other materials (e.g., PEI or siloxanes) to achieve certain material characteristics, including zero salt rejection, enhanced dehumidification performance, more size and charge selectivity to mention but a few.

### Future Outlook on Applications With Ultrathin Cellulose Films

Overall, utilizing the inherent structure of cellulose within the thin films for materials applications is an approach still in its infancy. There are no commercial applications at present but the current scientific literature gives indications as to where some of the business-related interests may lie in the near future. For example, clear proof-of-concept exists of functioning photoresists from cellulose and its derivatives. Cellulose based photoresist overcome several issues of conventional photoresists. For the preparation of photoresists, environmentally harmful and often toxic solvents such as chlorobenzene are used while the photoresist materials consist of persistent polymers that contribute to plastic pollution. Instead, biodegradable cellulose derivatives proposed so far for phototresists can be processed from ecosolvents such as ethyl acetate. European legislation will further push the replacement of conventional, non-biodegradable, non-renewable synthetic polymers by biobased greener solutions in the next decade and this will certainly affect photoresists as well. Still, further developments are required to commercialize the technology into products, involving adhesion on various substrates, efficiency, durability and resistance in harsh environments for instance.

Similar issues abound with membrane technology: organic solvents are generally required for preparation of the present commercial membranes and this could be omitted particularly if water-based nanocellulose dispersions are used for membrane manufacture. Ultimately, however, the performance of cellulose thin films will dictate their pliability in relevant applications. Concern about the dimensional stability of cellulose films under water and humidity, for example, is one of the factors that could limit its usage.

However, the specific response of cellulose to water and humidity may be seen as an advantageous issue from materials perspective. Recent work on fundamental understanding of the cellulose-water interactions within ultrathin films underline this relationship (Kontturi et al., 2013; Niinivaara et al., 2015, 2016; Tammelin et al., 2015; Reid et al., 2016; Hakalahti et al., 2017). Indeed, the native functions of cellulose may be utilized for specific functionalities in the future. Humidity sensors or various actuators based on humidity would be obvious examples for potential applications. There is, however, room also for more sophisticated functional materials based on water/humidity response of cellulose in thin films in the realm of, e.g., smart material solutions.

Biomedical applications of cellulose based thin films have been shown to have a huge potential as well. Here, several proof of concepts from various groups do exist that propose the detection of biomolecules such as DNA and proteins. Proteins seem to have the largest potential for commercialization in the field of enzyme immobilization and antibody detection. Antibody detection is a crucial point when it comes to blood typing and it was already shown that IgG can be effectively detected on such films. Here the combination of patterned cellulose thin films for in combination with microfluidics could be an opportunity to develop new products.

## Conclusion

This review has shown how the focus on cellulose films has recently shifted to another direction. Ultrathin films of cellulose were previously perceived mainly as tools for experimental modeling, by applying them as model films to probe and interpret fundamental physico-chemical phenomena occurring with cellulose-based materials. Although the modeling aspect is still important, utilization of these films in materials applications has become an equally important activity. This aspect is still in its infancy, but we see a significant potential in the approach where the functional features of cellulose in its native environment could be utilized for its applications within ultrathin films.

## Author Contributions

EK wrote the Introduction and Conclusions as well as the section on Film Deposition. SS was largely responsive for the section on Materials Applications.

### Conflict of Interest Statement

The authors declare that the research was conducted in the absence of any commercial or financial relationships that could be construed as a potential conflict of interest.
